# Supplementary material for: Replica Exchange Nested Sampling
Source: J Chem Theory Comput. 2025 Jul 24;21(15):7304–19. doi: 10.1021/acs.jctc.5c00588 (PMC12355697; doi:10.1021/acs.jctc.5c00588)
Supplement: Supplementary file 1 [file ct5c00588_si_001.pdf]

Replica exchange nested sampling

Supplementary Material

N. Unglert,[1] L. B. Pártay,[2] and G. K. H. Madsen[1,*]

[1]*Institute of Materials Chemistry, TU Wien, 1060 Vienna, Austria*

[2]*Department of Chemistry, University of Warwick, Coventry CV4 7AL, UK*

(Dated: June 13, 2025)

TOY MODEL

Additional combinations of K and L parameters

In the main article we presented the expected value of observables $a(P,T)$ and $C_P(P,T)$ for both RENS and independent NS calculations for $L = 10$ and $K = 10, 20$ and 50 (see Figure 6). In Fig. S1a and b we include additional results, calculated using longer MCMC walks of $L = 20$ and 50, used in combination with different number of walkers.

Replica exchange swap acceptance rates

In Fig. S1c, we present the RE swap acceptance probabilities for an example run over the course of the simulation.

The observed acceptance rates in RENS for atomistic systems follow a characteristic pattern: they start high and gradually decline to near zero as the simulation approaches the ground state. This behavior reflects the evolving nature of the underlying distributions, which dynamically change throughout the run. Toward the end of the NS process, the likelihood-constrained prior distributions typically no longer overlap.

Unlike classical parallel tempering, where neighboring replicas' temperature intervals can be optimized based on a static average swap acceptance rate, the shifting distributions in RENS make it impossible to define fixed, optimal replica intervals. Since NS requires each individual simulation to operate on a fixed likelihood surface, the replica intervals must remain constant throughout a RENS run. As a result, vanishing acceptance rates toward the end of the sampling process are in general inevitable. However, in many cases, swaps may no longer be necessary at this stage, as all relevant modes are already occupied. We emphasize that swap acceptance rates serve as a key metric for optimizing parameters in successful RENS simulations. In the following, we illustrate this in detail using the more practical case of the periodic LJ system.

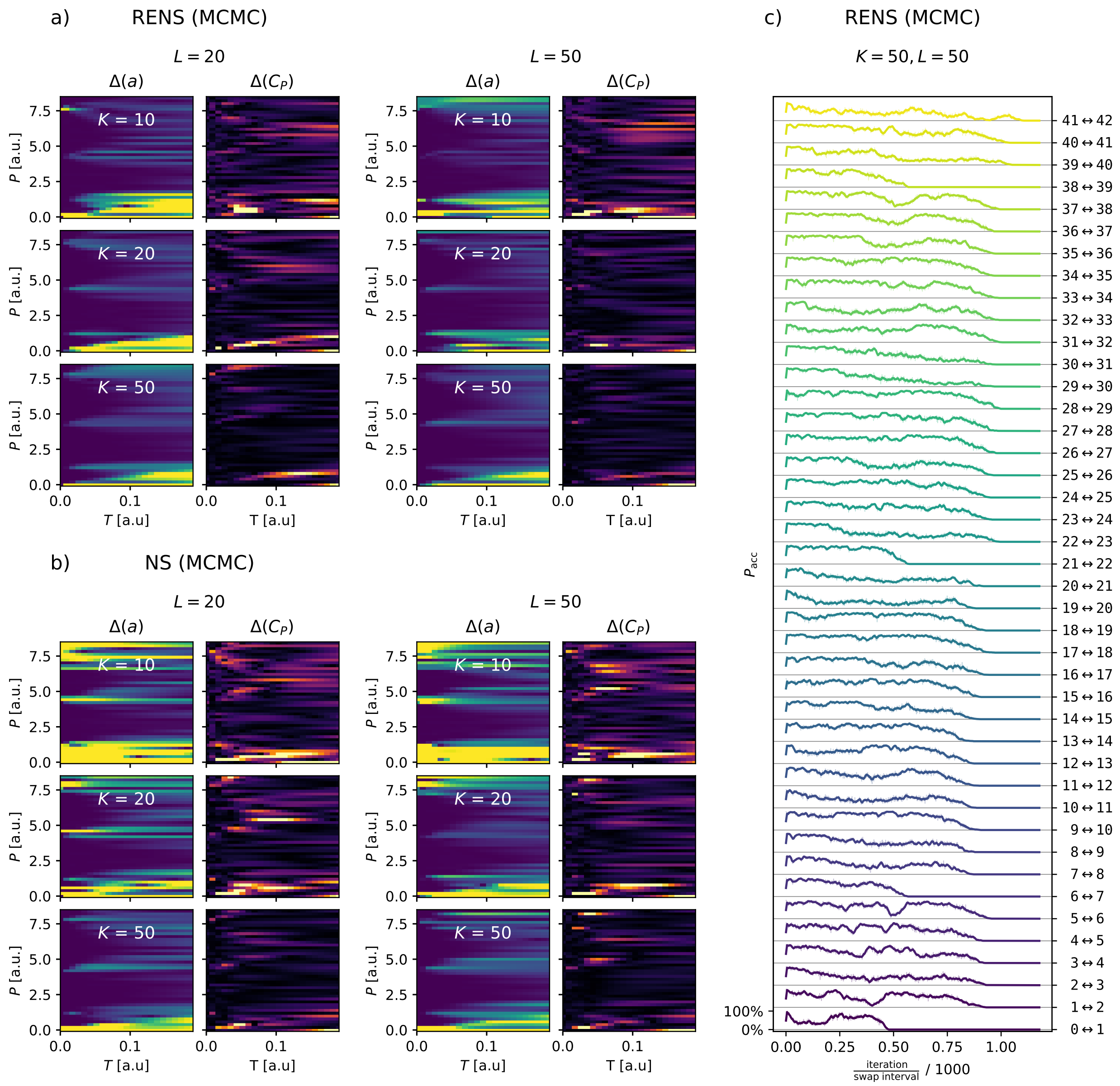

FIG. S1. Deviation of the thermodynamic expectation values of the lattice parameter a and the constant pressure heat capacity C_P from a converged reference simulation of the toy model with $M = 43$. Results are shown for $L = 20, 50$ and $K = 10, 20, 50$. a) for RENS using MCMC b) for independent NS using MCMC c) RE swap acceptance rates for an exemplary $K = 50$, $L = 50$ RENS simulation.

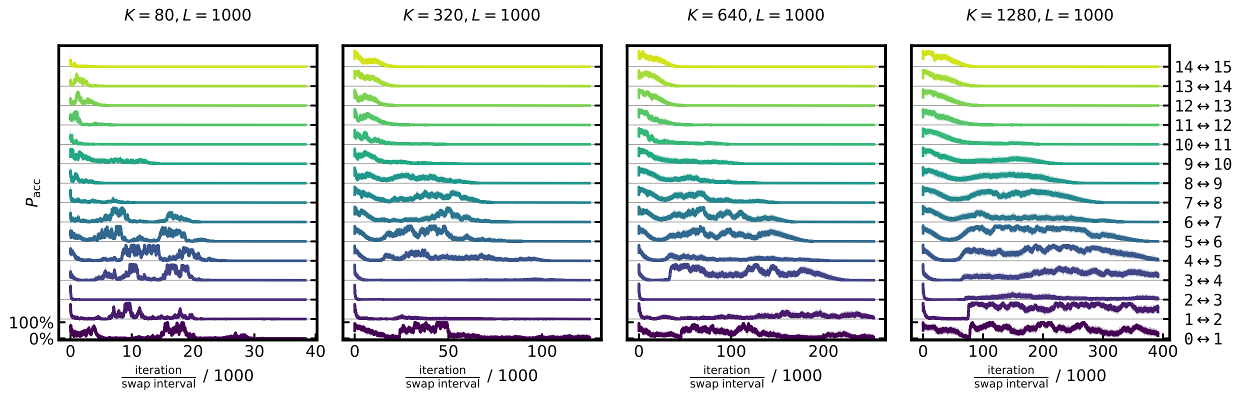

FIG. S2. Swap acceptance rates corresponding to the RENS simulations of periodic Lennard-Jones with $M = 16$ shown in Fig. 5b for a set of $K = 80, 320, 640, 1280$ at fixed $L = 1000$.

LENNARD-JONES

Replica exchange swap acceptance rates

To complement the observations on Fig. 7 in the main article, here we present a detailed analysis of the RE swap acceptance rates in case of the periodic Lennard-Jones RENS simulations. Fig. S2 shows the RE swap acceptance rates corresponding to the RENS simulations shown in Fig. 7b. A similar trend is observed, with the acceptance rates gradually vanishing towards the end of the sampling.

To better understand how the RE swap acceptance rates change in the course of our calculations, we performed a RENS simulation with $K = 80$ and $L = 1000$, during which we saved the walker populations at various iterations. This allows us to analyse the properties and the distribution of walkers at various stages of the sampling, thus the features which fundamentally determine the swap acceptance probabilities. The RE swap acceptance rates for this run, shown in Fig. S3, exhibit similar trends as previously discussed in Fig. S2.

Two key features are immediately apparent. First, the acceptance rate for the $(1 \leftrightarrow 2)$ swaps rapidly declines and remains zero after only a few thousand iterations. Second, the acceptance rates for high-pressure runs are generally very low and also decay quickly. To investigate these further, we examine the volume distributions of the walker populations for the four lowest-pressure replicas at different iterations up to $i = 70 \times 10^3$, as shown in Fig. S4a. Around iteration 15000, the distributions of replicas 2 and 3 begin to diverge from those of replicas 0 and 1, leading to a loss

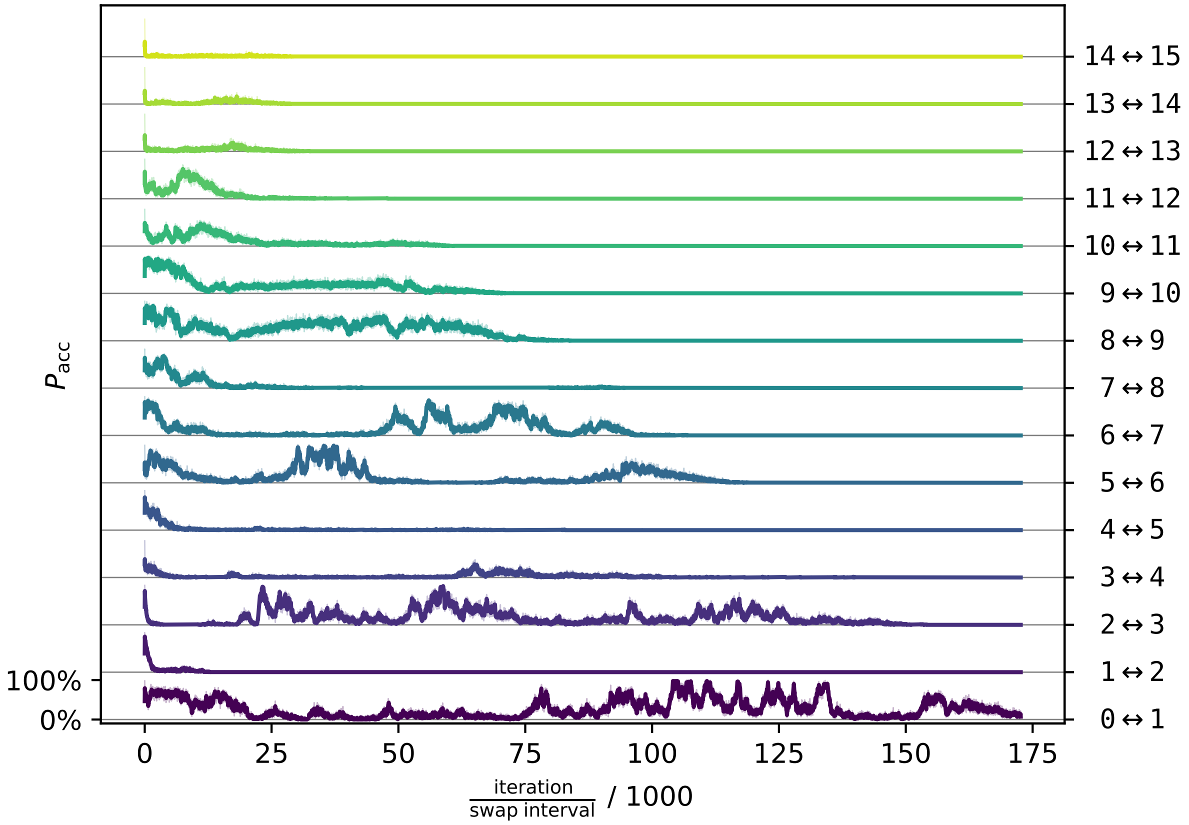

FIG. S3. RE swap acceptance rates for a RENS run of the periodic Lennard-Jones system with $K = 80$, $L = 1000$ and $M = 16$.

of overlap between replicas 1 and 2. As a result, the swap acceptance rate for $(1 \leftrightarrow 2)$ vanishes. Notably, even though the volume distributions start to overlap again around iteration 55000, the acceptance rates remain zero. This is due to the volume becoming an unreliable order parameter in this higher-density regime.

A similar reasoning explains the low acceptance rates for high-pressure replica transitions. Figure S4b shows the evolution of the volume distributions for the six highest-pressure replicas. Due to the large pressure differences imposed by the logarithmic pressure scale, the volume distributions rapidly split into distinct equilibrium volumes. While replicas 10 and 11 maintain some overlap up to $i = 70000$, the volume distributions of replicas 11 to 15 lose overlap much earlier, perfectly correlating with the acceptance rates observed in Fig. S3. This behavior is a direct consequence of the increasing influence of the PV term at higher pressures, leading to significant

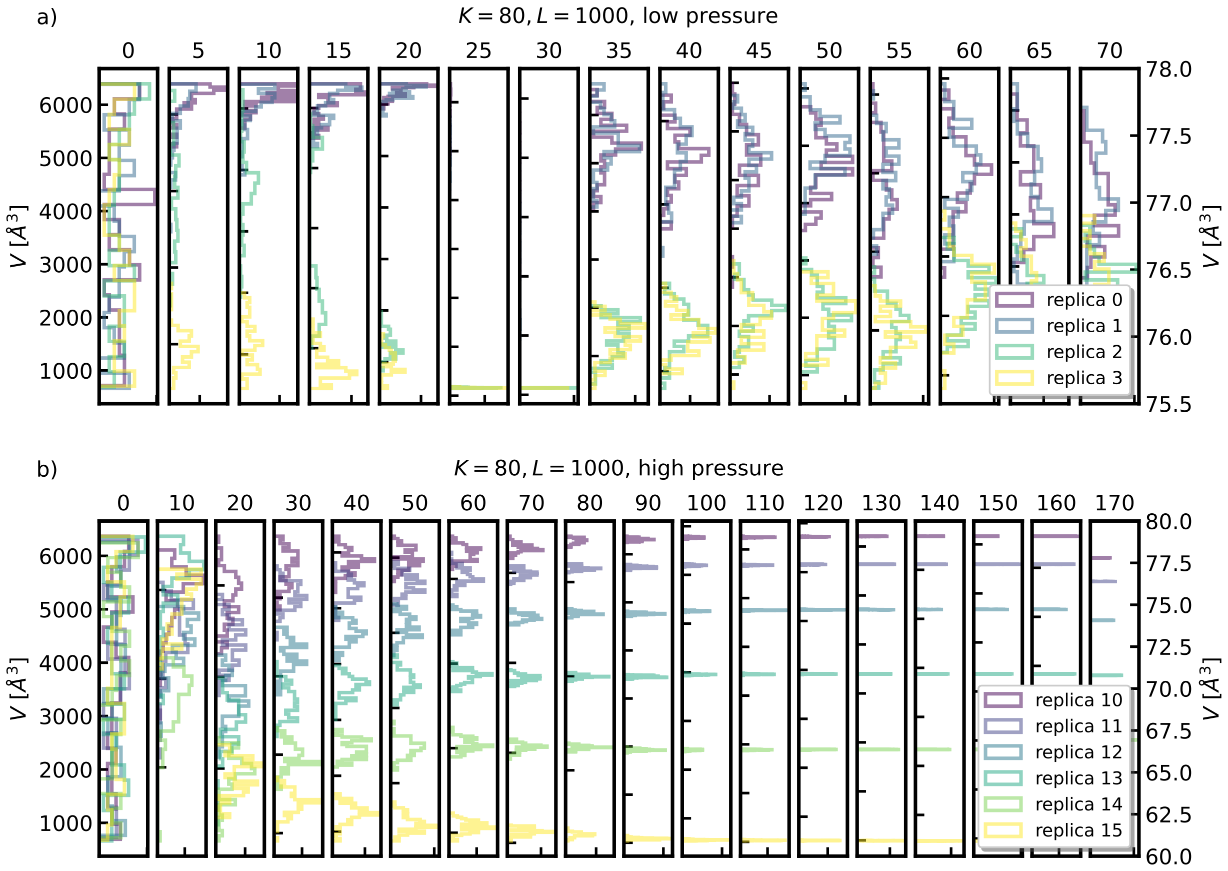

FIG. S4. Volume distribution of walker populations for different replicas of a RENS simulation for periodic Lennard-Jones with $K = 80$, $L = 1000$ and $M = 16$ at various iterations. Note, that the range of the volume axis changes with each iteration, accounting for the volume compression in the course of the simulation.

differences in the enthalpy surfaces between replicas.

These observations highlight two critical factors that negatively impact swap acceptance rates and, consequently, the efficiency of RENS simulations: (i) walker distributions loosing overlap during the run and (ii) neighboring replicas with posterior distributions that are too different such that walker distributions never overlap properly. Both issues can be systematically addressed. By construction of the NS algorithm, the evolution of the walker distribution with respect to iteration i behaves stochastically and is strongly dependent on K. Extreme low values of K can lead to severe shrinkage of the configuration space during a single iteration and, consequently, in the walker distribution. This increases the likelihood of losing overlap between the distributions permanently. This issue can be easily mitigated by choosing larger K values, as seen in Fig. S2, where increasing K reduces the frequency of vanishing swap acceptance rates.

For atomistic systems, the problem of highly dissimilar posterior distributions can be directly controlled by adjusting the difference in thermodynamic forces (in this case, pressure). Reducing these differences improves overlap between walker distributions and enhances swap acceptance. However, it is important to note that even when swap acceptance rates vanish between two neighbor replicas, the RENS framework as a whole does not necessarily break down. Instead, the simulation effectively transitions into multiple independent RENS simulations around the affected pressure, or in the extreme case where all swap acceptances drop to zero, it simply reduces to a set of independent NS simulations.

In conclusion, swap acceptance rates serve as a crucial diagnostic tool when conducting RENS simulations. Their detailed analysis provides valuable information on the functionality of the RENS approach and helps guide the selection of optimal NS parameters (specifically K) as well as replica intervals.

JAGLA

Additional simulations

Figure S5 compares three simulations conducted with $K = 250$ and $L = 1000$, which corresponds to one-fourth of the computational effort of the $K = 1000$, $L = 1000$ simulations presented in the main article (see Fig. 8). Figures S5a and b show results from an independent NS and a RENS simulation, respectively, performed at 28 pressures ranging from 0.01 to 0.28. While the independent NS results closely resemble the limited information obtained with $K = 1000$, the RENS simulation appears to be significantly affected by the reduction of the sampling resolution. Although the heat capacity suggests an improvement over the independent NS, the density and order parameter indicate that the $Ia\bar{3}d$ phase and its transition to the low-temperature $Fddd$ structure are not sufficiently sampled. To further investigate this, Fig. S5c presents an additional RENS simulation with the same NS parameters of K and L, but with an expanded pressure range of 56 pressure values from 0.01 to 0.56. With this adjustment, the total number of walkers across replicas increase, and the results closely match those from the $K = 1000$ simulation.

These findings suggest that in some cases, adding replicas at external conditions beyond the original region of interest should be considered, as they can significantly enhance the resolution of the sampling and thus the performance of RENS.

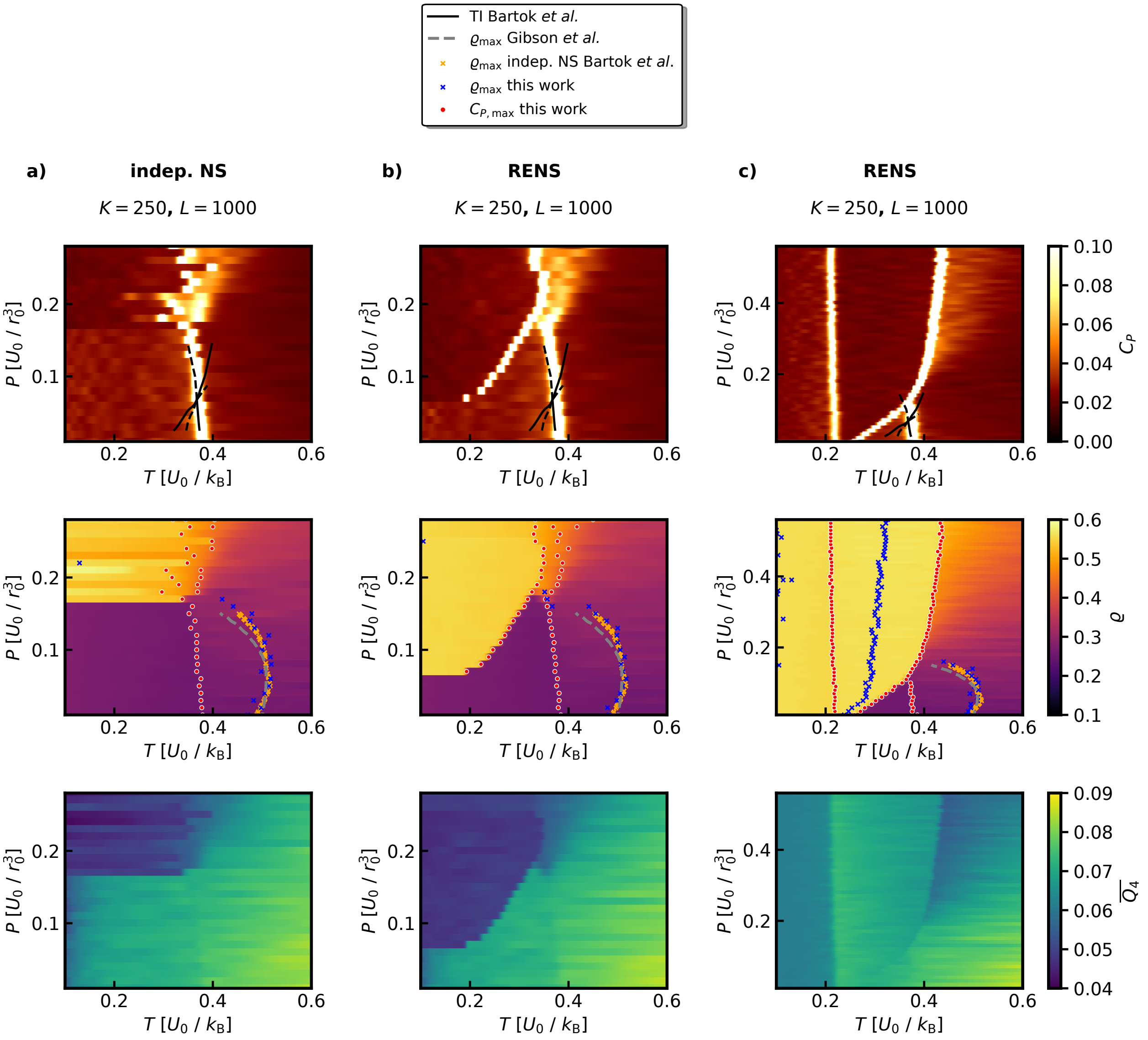

FIG. S5. Pressure and temperature dependent expectation values from NS simulations using $K = 250$ and $L = 1000$ of the 64 particle Jagla system. a) for independent NS at $M = 28$ pressures from 0.01 to 0.28 b) for RENS at $M = 28$ pressures from 0.01 to 0.28 c) for RENS at $M = 56$ pressures from 0.01 to 0.56 Top: Heat capacity C_P, black lines show coexistence lines computed using thermodynamic integration (TI) taken from Ref. [1], dashed lines indicate extensions into metastable regions. Middle: density ρ compared to liquid density maxima from independent NS [1] as well as Ref. [2]. Bottom: Steinhardt $\overline{Q_4}$ parameter.

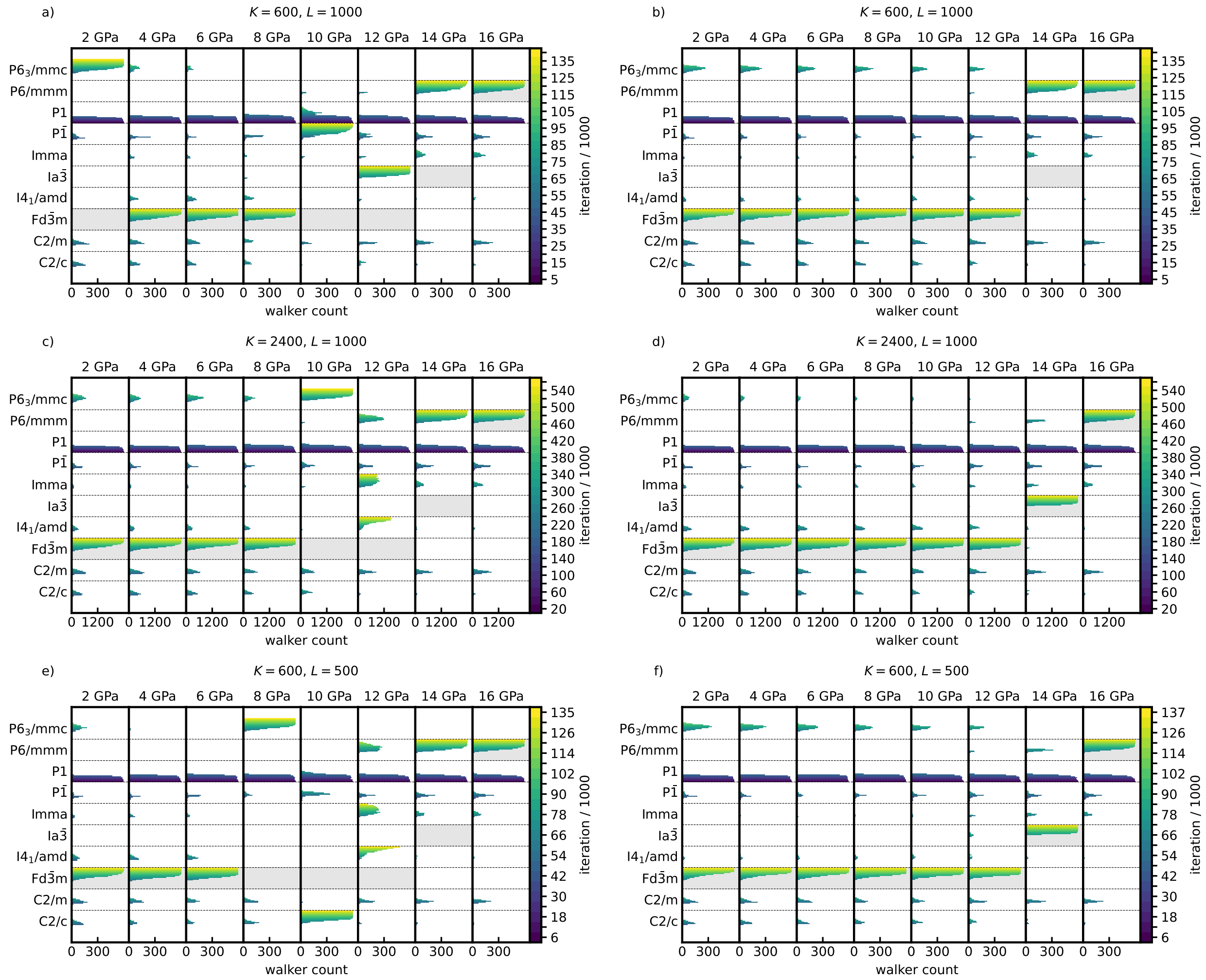

FIG. S6. Analysis of the basins that are explored during different nested sampling runs with varied number of walkers K and walk length L at $M = 8$ pressures. Left column show independent NS runs, right column shows RENS runs. Expected ground state phases from EoS computations (compare Fig. 9) are shaded in grey. The colorscale represents the population at a given iteration i.

SILICON

Additional combinations of K and L parameters

Here, we present the symmetry analysis for an additional combination of (K, L) parameters discussed in the main article in the context of Fig. 10 and Fig. 11.

Figure S6a and c display independent NS simulations with $L = 1000$, where the number of walkers is increased from $K = 600$ to $K = 2400$, respectively. While this leads to a slight

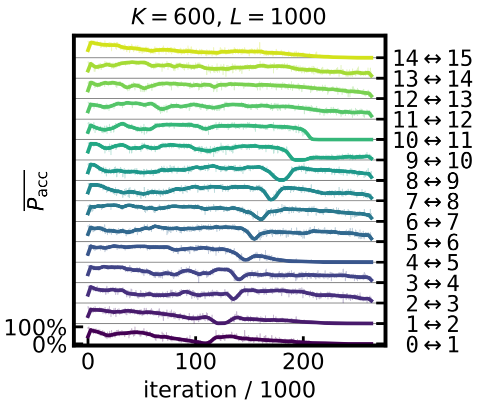

FIG. S7. Averaged swap acceptance rates for the RENS simulation of silicon using $K = 600$, $L = 1000$ and $M = 16$, shown in main text in Fig. 13c. Note, that in contrast to the previously shown acceptance rates P_{acc}, $\overline{P_{\text{acc}}}$ indicate the averaged swap acceptance rates over the last $n_{\text{monitor}} = 100$ iterations.

improvement compared to the $K = 100$ simulation shown in the main text, even at $K = 2400$, the intermediate pressure region remains mispredicted. In contrast, the RENS simulations exhibit minimal differences compared to the $K = 100$ RENS simulation from the main text, reinforcing the conclusion that RENS achieves convergence already at $K = 100$, whereas independent NS fails to do so within the shown parameter range.

Swap acceptance rates

Acceptance rates for the $K = 600$, $L = 1000$ RENS simulation (compare Fig. 13c and d) on a finer pressure grid of $M = 16$ pressures between 8 and 20 GPa are shown in Fig. S7.

* georg.madsen@tuwien.ac.at

[1] A. P. Bartók, G. Hantal, and L. B. Pártay, Insight into Liquid Polymorphism from the Complex Phase Behavior of a Simple Model, Phys. Rev. Lett. **127**, 015701 (2021).

[2] H. M. Gibson and N. B. Wilding, Metastable liquid-liquid coexistence and density anomalies in a core-softened fluid, Phys. Rev. E **73**, 061507 (2006).
